# Genetic diversity of *Plasmodium falciparum* among school-aged children from the Man region, western Côte d’Ivoire

**DOI:** 10.1186/1475-2875-12-419

**Published:** 2013-11-15

**Authors:** Sarah E Mara, Kigbafori D Silué, Giovanna Raso, Simon P N’Guetta, Eliézer K N’Goran, Marcel Tanner, Jürg Utzinger, Xavier C Ding

**Affiliations:** 1 Unité de Formation et de Recherche Biosciences, Université Félix Houphouët-Boigny, 01 BP V34 Abidjan 01, Côte d’Ivoire; 2 Département Environnement et Santé, Centre Suisse de Recherches Scientifiques en Côte d’Ivoire, 01 BP 1303 Abidjan 01, Côte d’Ivoire; 3 Department of Epidemiology and Public Health, Swiss Tropical and Public Health Institute, P.O. Box, CH-4002, Basel, Switzerland; 4 University of Basel, P.O. Box, CH-4003, Basel, Switzerland

**Keywords:** *Plasmodium falciparum*, *msp2*, Genetic diversity, PCR-RFLP, Côte d’Ivoire

## Abstract

**Background:**

The genetic diversity of *Plasmodium falciparum* allows the molecular discrimination of otherwise microscopically identical parasites and the identification of individual clones in multiple infections. The study reported here investigated the *P. falciparum* multiplicity of infection (MOI) and genetic diversity among school-aged children in the Man region, western Côte d’Ivoire.

**Methods:**

Blood samples from 292 children aged seven to 15 years were collected in four nearby villages located at altitudes ranging from 340 to 883 m above sea level. Giemsa-stained thick and thin blood films were prepared and examined under a microscope for *P. falciparum* prevalence and parasitaemia. MOI and genetic diversity of the parasite populations were investigated using *msp2* typing by polymerase chain reaction-restriction fragment length polymorphism (PCR-RFLP).

**Results:**

*Plasmodium falciparum* prevalence and parasitaemia were both found to be significantly lower in the highest altitude village. Genotyping of the isolates revealed 25 potentially new *msp2* alleles. MOI varied significantly across villages but did not correlate with altitude nor children’s age, and only to a limited extent with parasitaemia. An analysis of molecular variance (AMOVA) indicated that a small, but close to statistical significance (*p* = 0.07), fraction of variance occurs specifically between villages of low and high altitudes.

**Conclusions:**

Higher altitude was associated with lower prevalence of *P. falciparum* but not with reduced MOI, suggesting that, in this setting, MOI is not a good proxy for transmission. The evidence for partially parted parasite populations suggests the existence of local geographical barriers that should be taken into account when deploying anti-malarial interventions.

## Background

The widespread genetic diversity of *Plasmodium falciparum* populations plays an important role on several aspects of parasite biology, and consequently on the efficacy of anti-plasmodial interventions. For example, polymorphic elements and genetic plasticity contribute to modulate malaria morbidity and mortality and play a key role in *P. falciparum* immune evasion and the parasite’s adaptation to evolving environmental conditions such as drug pressure [1,2]. This highly polymorphic nature can also be harnessed to study the structure and dynamics of parasite populations (see, for example, references [3,4]) and molecular investigations in epidemiological studies and clinical trials, allowing discrimination between re-infection and recrudesce [5]. This is typically achieved by typing one or more of three highly polymorphic genes: *msp1* (*merozoite surface protein 1*), *msp2* (*merozoite surface protein 2*), and *glurp* (*glutamate-rich protein*), which encode antigenic proteins of poorly characterized functions [6].

Studies pertaining to the genetic diversity of *P. falciparum* have been essentially comparing either very distant locations (e.g. intercontinental or spread within one or several countries, see [7-11]), or a small number of epidemiologically very distinct locations [12-15] and reported observations on the complex interplay between transmission intensity, the multiplicity of infection (MOI) with *Plasmodium* and the genetic structure of local parasite populations. High transmission settings have been frequently, but not always, associated with greater genetic diversity and MOI [16,17].

**Figure 1:**
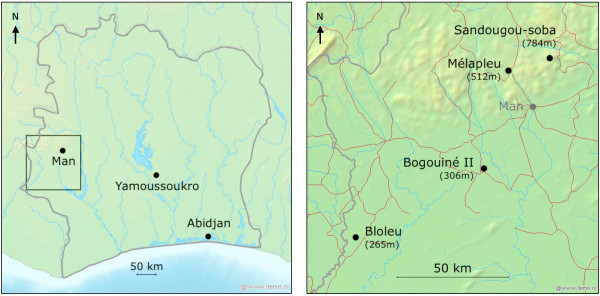
**Study sites location.** Four villages located in the Man region, western Côte d’Ivoire, were selected for this study. Bogouiné II and Bloleu are in a plain at relatively low altitude (340 and 346 m above sea level, respectively). Mélapleu and Sandougou-soba are in a mountainous area at relatively high altitude (529 and 883 m, respectively). Maps have been generated using GPS Visualizer [24].

A large study of the demographic, environmental and socioeconomic risk factors associated with malaria, schistosomiasis and soil-transmitted helminthiasis was previously conducted in 57 villages of the mountainous region of Man in western Côte d’Ivoire [18-21]. The local topography is an extension of the Fouta Djallon highland area and is characterized by rounded mountains in the north, ranging in altitudes from 200 to 1,300 m above sea level, and an abrupt transition to plains in the south. The area is hyperendemic for *P. falciparum* infection and polyparasitism (concurrent infection with different species of helminths, intestinal protozoa and *Plasmodium*) is very common [22]. In the work presented here, the MOI with *P. falciparum* is further explored. Additionally, the genetic diversity of the local *P. falciparum* populations is assessed for a subset of the previously collected samples. Specifically, four villages located in close proximity to each other but at various altitudes have been selected (Figure 1). Emphasis is placed on a small-scale comparison of *P. falciparum msp2* allelic diversity in close but geographically contrasted areas of western Côte d’Ivoire. The importance of the findings in the context of the current malaria elimination and eradication agenda [23] is discussed.

## Methods

### Ethical consideration

The study protocol was approved by the institutional research commission of the Swiss Tropical and Public Health Institute (Basel, Switzerland) and the Centre Suisse de Recherches Scientifiques en Côte d’Ivoire (Abidjan, Côte d’Ivoire). Ethical clearance was obtained from the Ministry of Health in Côte d’Ivoire. Regional health and education authorities, village leaders (chief, school director and teachers) and parents of school-going children were informed about the purpose and procedures of the study. Written informed consent was obtained from parents/legal guardians, whereas children assented orally. Participation was voluntary and children could withdraw anytime without further obligations.

### Study sites and population

The study sites are located in the Man region in western Côte d’Ivoire, where *P. falciparum* is hyperendemic [21]. It is a humid forest area with a dense river network that drains the entire region [25]. The northern part of the study area is mountainous whereas the southern part is a plain.

Between October 2001 and February 2002, 57 villages of this area were investigated to study the spatial distribution of infections with *Plasmodium* spp. [20,21], *Schistosoma mansoni*[18,25], and hookworm [19] among school-aged children. For the present study, a subset of four villages was selected, namely (i) Bogouiné II (geographical coordinates: 7 09.000 N latitude, 7 45.000 W longitude, 340 m above sea level); (ii) Bloleu (6 52.250 N, 8 16.333 W, 346 m); (iii) Mélapleu (7 32.800 N, 7 39.000 W, 529 m); and (iv) Sandougou-soba (7 35.833 N, 7 28.916 E, 883 m). The villages are located in a close range, at 120 km maximal distance, but in contrasting geographical settings with a maximal altitude difference of approximately 540 m. Mélapleu and Sandougou-soba are located northwest of Man, in the mountainous area, whereas Bogouiné II and Bloleu are located south of Man in a plain (Figure 1).

As described in detail elsewhere [18], all primary school children attending grades 3-5 were invited to participate. The study population in the four selected villages consisted of 292 school-going children, aged between seven and 15 years. Anti-malarial treatments were given to children with malaria-related symptoms (e.g. headache) and axillary temperature ≥37.5°C. Children found with helminth eggs in their stool were given anthelminthic drugs (i.e. praziquantel, 40 mg/kg against *S. mansoni*; albendazole, 400 mg against soil-transmitted helminths) [18,19].

### Sampling and molecular analysis

Finger-prick whole blood samples were obtained from each child. Thick and thin blood films were prepared on microscope slides and air-dried. Additionally, approximately 200 μl of blood was collected into microtainer tubes. Samples were transferred to a laboratory in the town of Man. Slides were stained with 10% Giemsa and forwarded to a laboratory in Abidjan, where they were examined under a microscope by experienced laboratory technicians for *Plasmodium* species identification and parasitaemia, assuming a count of 8,000 leukocytes per μl of blood [20,21]. Microtainers were stored at −20°C pending molecular analyses.

Blood samples were thawed on ice and used for *Plasmodium* DNA extraction, *msp2* nested polymerase chain reaction (PCR) amplification and analysis by restriction fragment length polymorphism (RFLP), as described elsewhere [26-28]. Briefly, 10 μl of whole blood were washed twice in cold sodium phosphate buffer (5 mM, pH 8.0), boiled for 10 min in 50 μl of sterile ddH_2_O and centrifuged at 14,000 *g* for 10 min. Next, 5 μl of the supernatant were used for the *P. falciparum msp2* nested PCR reactions. The presence of PCR amplification products was determined using ethidium bromide stained 1% agarose gel photographed under UV illumination. PCR amplification products were digested with HinfI restriction enzymes. Restriction digest products were resolved on a 10% acrylamide gel subsequently stained with ethidium bromide and photographed under UV illumination.

The size of restriction fragments was determined using the ImageJ software [29] together with the MolWT macro (supplied by PHASE GmbH; Luebeck, Germany). Allelic determination was performed as recommended previously and using 10 bp bins for the 3D7 variable fragment [28].

### Statistical analysis

The data were entered into Microsoft Excel 2007 and analysed using Prism version 6.0c or GenAlEx version 6.5 [30,31]. The number of effective alleles was calculated as [1/Σ*pi*^2^], the Shannon’s information index as [−1*Σ(pi*Ln(*pi*)], the haploid genetic diversity as [1-Σ*pi*^2^], and the theoretical probability of two clones to share the same genotype as [Σ*pi*^2^], where n is the sample size and *pi* the frequency of allele *i*.

## Results

### *Plasmodium falciparum* prevalence and parasitaemia

Overall, 292 blood samples from four selected villages were analysed by PCR for the presence of *P. falciparum* parasites. A total of 241 samples were found positive, resulting in a prevalence of 82.5%, which is slightly higher than the prevalence originally determined by microscopy, at 78.8% (see Additional file 1). For all subsequent analyses, the 27 samples found positive by PCR but negative by microscopy, were assigned a parasitaemia of four parasites/μl of blood. The prevalence in Bogouiné II, Bloleu and Mélapleu were 87.0%, 89.7% and 88.5%. A significantly lower prevalence was found in Sandougou-soba (57.4%), the village at the highest altitude (*p* < 0.01, χ^2^-test).

Parasitaemia, as determined by microscopy and corrected as mentioned above, was also found to be significantly different across villages (*p* = 0.039, Kruskal-Wallis test), with geometric means (GMs) ranging from 80.1 to 331.6 parasite/μl of blood (Table 1). Similarly to prevalence, the lowest parasitaemia GM was noted in the village located at the highest altitude, Sandougou-soba.

**Table 1:** ***Plasmodium falciparum *** **prevalence and parasitaemia at the study sites**

**Village**	**Altitude (m above sea level)**	**Sampling date (dd.mm.yyyy)**	**n**	**Age**^**a**^	***P. falciparum-*infected (%)**^**b**^	**Parasitaemia (95% CI)**^**c**^
Sandougou-soba	883	13.12.2001	54	10.2 (1.5)	31 (57.4)	80.1 (31.3-204.7)
Mélapleu	529	29.11.2001	78	10.1 (1.9)	69 (88.5)	207.6 (135.1-319.3)
Bloleu	346	07.02.2002	68	10.0 (1.1)	61 (89.7)	227.3 (138.9-371.8)
Bogouiné II	340	29.01.2002	92	10.1 (1.1)	80 (87.0)	331.6 (220.8-498.0)

^a^Average in years (SD).

^b^Number of *P. falciparum*-infected children, *p* < 0.01 (χ^2^).

^c^Geometric mean of parasites/μl of blood, *p* = 0.039 (Kruskal-Wallis test).

### *msp2* genotyping and multiplicity of infection

**Figure 2:**
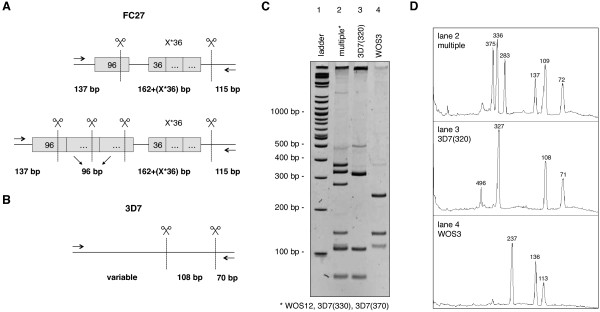
***msp2 *genotyping by PCR-RFLP.** HinfI restriction digest pattern of **(A)** FC-27 type alleles and **(B)** 3D7-type alleles. The sizes of fixed fragments are indicated, repetitive elements are indicated as grey boxes with sizes in bp. **(C)** Typical polyacrylamide gel electrophoresis result. Lane 2 contains a mixture of one FC27-type allele (WOS12) and two 3D7-type allele (330 and 370). Lanes 3 and 4 contain a single *msp2* allele. **(D)** ImageJ fragment size analysis of the gel displayed in panel C. The 3D7 108 bp fragment and the FC27 115 bp fragment co-migrate as a single peak in case of mixed sample (lane 2).

In order to better define the epidemiological profiles of the four study villages, a detailed *msp2* genotyping analysis was carried out, using RFLP on the 241 PCR-positive samples. Of note, *msp2* alleles are generally grouped in two distinct families, the FC27 type and the 3D7 type, which are characterized by the presence of specific repetitive elements. Moreover, a large diversity of alleles exists within each family type and these can be readily distinguished by analysing the size of fragments obtained after a HinfI digestion (Figure 2A, B). FC27-type alleles are characterized by two fragments of fixed size, 115 bp and 137 bp, together with a combination of fragments of variable size, depending on the specific pattern of repeats. 3D7-type alleles are characterized by two fixed size fragments of 70 bp and 108 bp and a third fragment of variable size [28]. A public domain image processing software was employed to determine the size of the HinfI fragments obtained from each sample after separation on polyacrylamide gels (Figure 2C, D) [29]. To evaluate the precision of the size determination method, the measures obtained with fixed-size fragments, that is the 115 bp and 137 bp fragments from FC27-type alleles and the 70 bp and 108 bp ones from 3D7-type alleles, were used. Altogether, the averages of the measured fragments did not deviate of more than 3 pb from the expected sizes, with standard deviations all under 4 bp (see Additional file 2). It was thus considered reasonable to pool fragments of variable size in bins of 10 bp. When possible, the nomenclature reported previously for specific FC27 alleles was used, whilst the 3D7 alleles are named according to the bin size of the variable fragment.

A total of 85 distinct *msp2* alleles, 45 of the FC27-type and 40 of the 3D7-type, were identified from the 241 blood samples analysed (Table 2). Two recombinant alleles were also identified (FC27 REC1 and FC27 REC2) and classified as FC27-type. Eighteen of the 45 FC27-type alleles identified appear to have not yet been reported elsewhere and some of these display insertion or deletion in the 137 bp fragment. Similarly, seven of the 40 3D7-types alleles display an unusually short variable fragment (<300 bp) or an additional 45 bp fragment. These 25 potentially newly identified *msp2* alleles have been named Cot 7 to Cot 31, following the nomenclature used for *msp2* allele previously identified in Côte d’Ivoire [32]. The details of the specific HinfI fragments are indicated in Table 3.

**Table 2:** ***msp2 *** **allele type**

**Village**	**n**	**Na**^**a**^	**FC27-type**	**3D7-type**	***P. falciparum *** **infection**	**Mean MOI (SD)**^**b**^
Sandougou-soba	31	39	14	25	72	2.32 (1.33)
Mélapleu	69	58	30	28	235	3.41 (1.75)
Bloleu	61	58	27	31	201	3.29 (1.72)
Bogouiné II	80	60	26	34	186	2.32 (1.38)
Total	241	85	45	40	694	2.88 (1.65)

^a^Number of distinct alleles.

^b^*p* < 0.001 (Kruskal-Wallis test on site-specific MOIs).

**Table 3:** **Details of potentially new *msp2 *** **alleles**

**Name**	**Family type**	**Hinf I fragment sizes**^**a**^			
Cot7	FC27	115	137	~244	
Cot8	FC27	115	137	~320	
Cot10	FC27	115	137	~228	
Cot11	FC27	115	137	~280	
Cot13	FC27	115	~131	~320	
Cot15	FC27	115	137	~191	
Cot14	FC27	115	~120	~227	
Cot17	FC27	115	137	~440	
Cot19	FC27	115	137	~396	
Cot20	FC27	115	137	~404	
Cot21	FC27	115	137	~454	
Cot22	FC27	115	137	~207	
Cot24	FC27	115	137	~430	
Cot25	FC27	115	137	~415	
Cot26	FC27	115	137	~357	
Cot29	FC27	115	137	~333	
Cot30	FC27	115	137	~180	
Cot31	FC27	115	~198	~237	
Cot9	3D7		70	108	~153
Cot12	3D7		70	108	~236
Cot16	3D7		70	108	~190
Cot18	3D7	~45	70	108	~490
Cot23	3D7		70	108	~180
Cot27	3D7	~45	70	108	~300
Cot28	3D7	~45	70	108	~460

^a^Exact sizes of fixed fragments are given, ~ indicates measured sizes.

Overall, 694 distinct *P. falciparum* infections were recorded from the 241 PCR-positive samples analysed. MOI, that is the number of clonal infection per individual, ranges from 1 to 8, with a mean of 2.88 (Table 2). The mean MOI were found to be significantly different across the study sites with values ranging from 2.32 to 3.42 (Table 2). This difference occurs between the geographically related villages and does not seem to correlate with the site-specific *P. falciparum* prevalence or mean parasitaemia. For instance, the highest parasitaemia GM (425.0) was observed in Bogouiné II, where the lowest mean MOI was found (2.32).

No link between MOI and participants’ age could be identified when considering the whole study population or each of the four villages separately (Table 4 and Additional file 3). A limited, but statistically significant, link between MOI and parasitaemia was observed (Table 4). MOI is significantly lower in the first parasitaemia quartile (four to 72 parasites/μl of blood) with a mean of 2.02, while it is comparable across the second, third and fourth quartiles with mean MOI values ranging between 3.12 and 3.23.

**Table 4:** Relationship between MOI and age or parasitaemia

**Variable**	**Stratification**	**n**	**MOI (SD)**	***p *** **value**
Age group	7-10 years	150	2.95 (1.76)	0.77^a^
	11-15 years	91	2.78 (1.47)	
Parasitaemia^b^	1st quartile	60	2.02 (1.13)	<0.001^c^
	2nd quartile	60	3.17 (1.80)	
	3rd quartile	61	3.23 (1.65)	
	4th quartile	60	3.12 (1.70)	

^a^Mann-Whitney test.

^b^Q1, Q2, and Q3 correspond to parasitaemia of 72, 440 and 740 parasites/μl of blood, respectively.

^c^Kruskal-Wallis test.

### Plasmodium falciparum genetic diversity

*Plasmodium falciparum* prevalence and parasitaemia, contrary to MOI, appear to be linked to some extent with the geographical location of the study sites. To further explore the specificity of the investigated sites, the site-specific *P. falciparum* genetic diversities were assessed.

**Figure 3:**
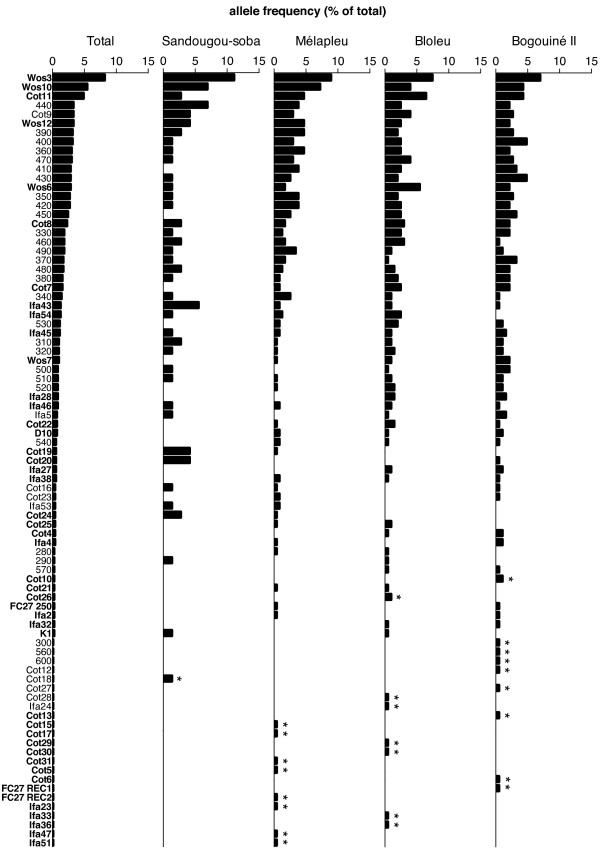
***msp2 *allele frequencies across study sites.** The overall (“Total”) and village-specific frequencies of each allele identified are indicated. Alleles in bold face are FC27-type and alleles in lightface are 3D7-type, * indicates private alleles.

Figure 3 displays the allelic frequencies observed at the respective study sites as well as when pooling all samples. The most prevalent allele in each village is the FC27-type allele WOS3, with a maximum local frequency of 11.1%. The second most prevalent allele in the two highest villages, Sandougou-soba and Mélapleu, is WOS10 (6.9% and 7.2%, respectively), while it is 3D7 (400) in Bogouiné II and the newly reported FC27-type allele Cot11 in Bloleu. These two alleles rank amongst the 18th and 9th most prevalent in the other locations, respectively. Between one and nine private alleles were identified in each village, but their local frequency did not exceed 1.4% (Figure 3). The lowest number of distinct *msp2* alleles (39) was observed in Sandougou-soba, which is likely due to the lower number of individual infections sampled in this village as compared to the others (72 *versus* 186 to 235) [33] (Table 5). Nevertheless, 60 different alleles were identified out of 186 clonal infections in the low altitude village Bogouiné II, while only 58 alleles were observed from 235 infections in the high altitude village Mélapleu. This suggests that high altitude villages might display a lower diversity than low altitude ones. This observation is further supported by the fact that the number of effective alleles, that is the theoretical number of equally frequent alleles needed to achieve a level of diversity equal to the one observed, is higher in Bogouiné II and Bloleu (low altitude) than in Sandougou-soba and Mélapleu (high altitude), despite the fact that the number of clonal infection sampled is lower in both of these villages as compared to Mélapleu (Table 5).

**Table 5:** ***Plasmodium falciparum *** **genetic diversity at the study sites**

**Village**	**n**	**Na**^**a**^	**Ne**^**b**^	**I**^**c**^	**h**^**d**^
Sandougou-Soba	72	39	23.78	3.427	0.958
Mélapleu	235	58	27.41	3.614	0.964
Bloleu	201	58	33.20	3.753	0.970
Bogouiné II	186	60	36.34	3.816	0.972
Total	694	85	n/a	n/a	n/a
Mean	n/a	n/a	30.18	3.652	0.966
SD	n/a	n/a	2.82	0.086	0.003

^a^Number of distinct alleles.

^b^Number of effective alleles.

^c^Shannon’s information index.

^d^Haploid genetic diversity.

Nevertheless, the haploid diversity indices were found to be very similar, with values close to 1 in all villages. Similarly, the Shanon’s information indices are relatively high, approaching or exceeding 3.5, in all populations. These two indices are indicative of a similarly high degree of diversity and evenness across study locations, suggesting that the local parasite populations investigated are genetically similar and display the same degree of diversity.

The specific geographic locations of the four study sites was further explored by grouping Sandougou-soba and Mélapleu as villages in the “mountainous” part of the Man region and Bogouiné II and Bloleu in the “plain” for a nested two-setting analysis of molecular variance (AMOVA) (Table 6). The *msp2* genetic diversity variations in the different villages were not found to be statistically different (ΦPT = 0), however, a low percentage of diversity variation occurring between the high and low altitude settings was found to be nearing statistical significance (ΦRT = 0.001, *p* = 0.07), suggesting that parasite populations might not be fully similar between these two settings.

**Table 6:** Two-setting nested analysis of molecular variance (AMOVA)

**AMOVA analysis**	**df**^**a**^	**MS**^**b**^	**% of total variation**	**ϕ**	***p *** **value**
Among settings^c^	1	0.619	0.13%	**ϕ** RT 0.001	0.070
Among populations within settings	2	0.418	0.00%	**ϕ** PR −0.001	0.785
Among all populations	80	0.486	99.87%	**ϕ** PT 0.000	0.275

^a^Degrees of freedom.

^b^Mean squared deviations.

^c^Sandougou-soba and Mélapleu are grouped in a “mountainous” setting, Bogouiné II and Bloleu in a “plain” setting.

## Discussion

A detailed molecular profiling and genetic diversity analysis of *P. falciparum* in four villages of a hyperendemic area of western Côte d’Ivoire is presented. The study sites were chosen for their relative closeness and contrasted geographical settings, with two villages located in a plain area and two in mountainous area. The *P. falciparum* prevalence, as determined by PCR, was found to be close to that previously reported by microscopy and above 85% in all villages except Sandougou-soba, the most elevated village, where it was measured to be 57.4% and where the parasitaemia GM was also found to be significantly lower. Importantly, the average age of participating children, a potential confounding factor for estimating *P. falciparum* prevalence and parasitaemia, is comparable from one village to another (Table 1), suggesting that geographical factors might be underlying these differences in the local parasitological pictures. This is in line with several previous studies reporting reduced malaria prevalence at higher altitude, with African highlands, at elevation of 1,500 m above sea level and higher, being generally malaria free but subject to epidemics [34-36]. Lower temperatures at high altitude reduce the development rates of both mosquitoes and *Plasmodium* parasites, preventing optimal transmission. At intermediate altitude, the fact that hilly environments are less susceptible to stagnant water bodies, which are potential mosquito breeding sites, might also play a role in locally reduced transmission [34]. It is, however, not clear whether these, or other factors, are responsible for the lower malaria prevalence and parasitaemia levels observed in Sandougou-soba.

The genetic diversity of the *P. falciparum* populations was characterized using *msp2* typing, whose higher discriminatory power, as compared to other polymorphic genes, is particularly relevant in hyperendemic areas [17]. The overall theoretical probability to have two clones sharing the same genotype in this study is of 3.4%, which is below the maximal value of 5% recommended by others [37], and warrants the use of *msp2* as a single marker for genotyping in this context. The genotyping methodology employed in the current study is based on electrophoresis in polyacrylamide gels coupled with computer-assisted fragment sizing using free software. This approach is well adapted to resource-limited settings and was found to be only marginally less precise than more costly methods based on capillary electrophoresis [17,38].

A total of 85 distinct *msp2* alleles were found in the current study and revealed 25 alleles not yet reported elsewhere, expending on a previous study of *msp2* genetic diversity in Côte d’Ivoire [32]. Two newly identified FC27 alleles, Cot13 and Cot14, display deletion in the 137 bp fragment, while an insertion is apparent in Cot31, possibly due the amplification of a 9 bp unit [39]. Some of the potentially new 3D7-type allele display an unusual fragment of approximately 45 bp (Cot18, Cot27 and Cot28), which might be similar to the 51 bp fragment previously observed in Tanzania [40]. Altogether, the limit in precision for fragment size determination does not exclude that some of these potentially new alleles are similar to the ones already described in the literature (e.g. Cot27 appears relatively similar to Ifa6; Cot31 to Ifa34) and sequencing would be required to univocally characterize these *msp2* alleles. Altogether, Wos3 is the most prevalent allele, similarly to what has been observed in south-central Côte d’Ivoire, and two of the newly described alleles in this earlier study were also observed here (Cot5 and Cot6) at low frequencies [32].

Apparent MOI is often used as surrogate for transmission, with higher multiplicity of infections observed in those areas where transmission is particularly high [16]. Consequently, correlations between MOI and parasitaemia or age have been reported [11,17,32,41-43]. In these studies, higher MOI are associated with higher parasitaemia and with younger age, possibly due to lower semi-immunity [44]. The complex interplay between MOI, parasitaemia, age and transmission is not fully understood and other studies failed to report correlations between these parameters, suggesting setting-specific relationships [9,45-47]. In the present investigation, significant differences in MOI averages across the four villages were observed, but with no apparent link with *P. falciparum* prevalence, parasitaemia, village altitude or sampling date (Table 1), the last being a potential confounding factor. A low multiplicity was found in Sandougou-soba, where the lowest prevalence and average parasitaemia were observed, but an equally low multiplicity was also apparent in Bogouiné II, where these parameters were comparatively higher. The factors involved that might explain local differences in MOI are currently not understood. When considering the four villages together, no clear relation was found between age and MOI and only a limited relation with parasitaemia. It appears that in the context of this study, no clear link can be made between MOI and *P. falciparum* prevalence or parasitaemia and, by extension, transmission or with altitude. This is contrasting with a previous study in Kenya, where highland infections display a significantly lower average MOI as compared with lowland areas [36]. The reasons for this difference are unknown and the intrinsic limitations of the current MOI measurements, which are by definition underestimating the real multiplicity of infection, due to clone fluctuation, parasite sequestration in deep vasculature, failure to distinguish different clones or true multiple infections by similar clones, might play a role. Nevertheless, these limitations would be expected to operate equally in all villages and therefore to bias absolute values but not relative comparison.

The *msp2* typing data were also used to evaluate the genetic diversity present in each of the study sites. It can be expected that lower transmission, observed at higher altitude, might result in lower genetic diversity. This hypothesis is supported by the lower number of effective alleles found at higher altitudes. This value can be biased by the sample size, so the most relevant comparison occurs between Bloleu (low altitude) and Mélapleu (high altitude), where an equal number of distinct alleles were observed out of a similar number of infections but with a marked difference in the number of effective alleles, that is 33.2 and 27.4, respectively. A similar trend can be observed, but to a much lower extent, when considering other measures of diversity, such as Shannon’s information index and the haploid genetic diversity, which is nearing its maximum value of one in all villages, similar to values previously observed in sub-Saharan Africa [11]. A low but close to significance degree of genetic variance is apparent between villages of plain and mountainous areas, suggesting the existence of a partially effective partition between the parasite populations of these locations. This is contrasting with a similar study conducted in Uganda, where no difference in strain distribution was observed when comparing villages located in an area of the same scale as the one considered here [46]. The current analysis is intrinsically limited by the fact that only one locus was investigated. It is conceivable that the typing of additional loci, including microsatellites, might have revealed stronger genetic differences. Moreover, the fact that *msp2* is under immune selection might partially mask the true genetic diversity of the populations investigated here. It would also be important to evaluate the temporal evolution of the genetic structures, as time has been identified as a diversity factor almost as important than geographical location in various transmission settings [15,48].

## Conclusion

The current study revealed marked differences in *P. falciparum* epidemiological and genetic factors within a context of hyperendemicity. Significant differences in prevalence and parasitaemia as well as signs of population partitions could be observed within a distance of approximately 120 km. The local topography in the Man region might partially govern these differences, suggesting the existence of geographical barriers susceptible to locally isolated parasite populations in Côte d’Ivoire. It would be interesting to measure the current genetic diversity in the four study villages in order to evaluate the evolution of the diversity over the past decade. The malERA consortium has highlighted the need for better tools and maps to guide malaria elimination [23], and the identification of natural or human-related barriers defining distinct or partially distinct populations will contribute to address this need. The identification of partially isolated parasite populations might be relevant to guide local malaria elimination intervention when a geographically limited area is targeted.

## Competing interests

This study received financial support from the Swiss Tropical and Public Health Institute and the Centre Suisse de Recherches Scientifiques en Côte d’Ivoire. The funders had no role in the study design, data collection and analysis, decision to publish, or preparation of the manuscript. All authors declare that they have no competing interests.

## Authors’ contributions

SEM performed the genotyping and statistical analyses and wrote the first draft of the manuscript. KDS collected the samples, conceived and performed the genotyping analyses and contributed to the writing of the manuscript. GR collected the samples, conceived the genotyping analyses, and contributed to the statistical analyses and to writing of the manuscript. SPN was responsible for the supervision of SEM. EKN, MT, and JU conceived the larger study within which the current genotyping profiling was pursued, were responsible for the supervision of KDS and GR and assisted in the revision of the manuscript. XCD conceived and performed the genotyping analyses and contributed to the statistical analyses and to the writing and revisions of the manuscript. All authors read and approved the final manuscript.

## Supplementary Material

Additional file 1 **PCR and microscopy derived prevalence. (A)***P. falciparum* detection by PCR and microscopy-based observation of thick smears. The overall higher prevalence detected by PCR (82.5% versus 78.8%) is due to 27 samples found positive by PCR and negative by microscopy, while 16 samples positive by microscopy were found negative by PCR. **(B)** Parasitaemia, as determined by microscopy, of the PCR negative (n = 16) and PCR positive (n = 214) samples. The geometric mean of the PCR negative samples is significantly lower than that of the PCR positive samples (70.3 versus 356.4, *p* < 0.01, unpaired one-tailed Welch’s t-test), suggesting slide reading errors or *P. falciparum* DNA degradation. Click here for file

Additional file 2 ***msp2 *** **genotyping precision. (A)** Average, standard deviation and range of the observed sizes of fixed-size msp2 restriction digest products. **(B)** Linear regression between the observed and expected sizes of the fixed-size msp2 restriction digest products. The average, standard deviation, miminum and maximum value are reported. Click here for file

Additional file 3 **Relation between MOI and age. (A)** Individual MOI grouped by age clusters (7 to 10 and 11 to 15 years old) in the four study villages. The red lines indicate the means and standard deviations. **(B)** The average MOIs are not significantly different in the two age clusters in each of the four study villages. Click here for file
