# Supplementary figures and images for: Genetic diversity of Plasmodium falciparum among school-aged children from the Man region, western Côte d’Ivoire
Source: Malar J. 2013 Nov 15;12:419. doi: 10.1186/1475-2875-12-419 (PMC3842749; doi:10.1186/1475-2875-12-419)

A

|                     | PCR positive<br>(%) | PCR negative<br>(%) | Total (%)   |
|---------------------|---------------------|---------------------|-------------|
| Microscopy positive | 214 (73.3)          | 16 (5.5)            | 230 (78.8)  |
| Microscopy negative | 27 (9.2)            | 35 (12.0)           | 62 (21.2)   |
| Total               | 241 (82.5)          | 51 (17.5)           | 292 (100.0) |

B

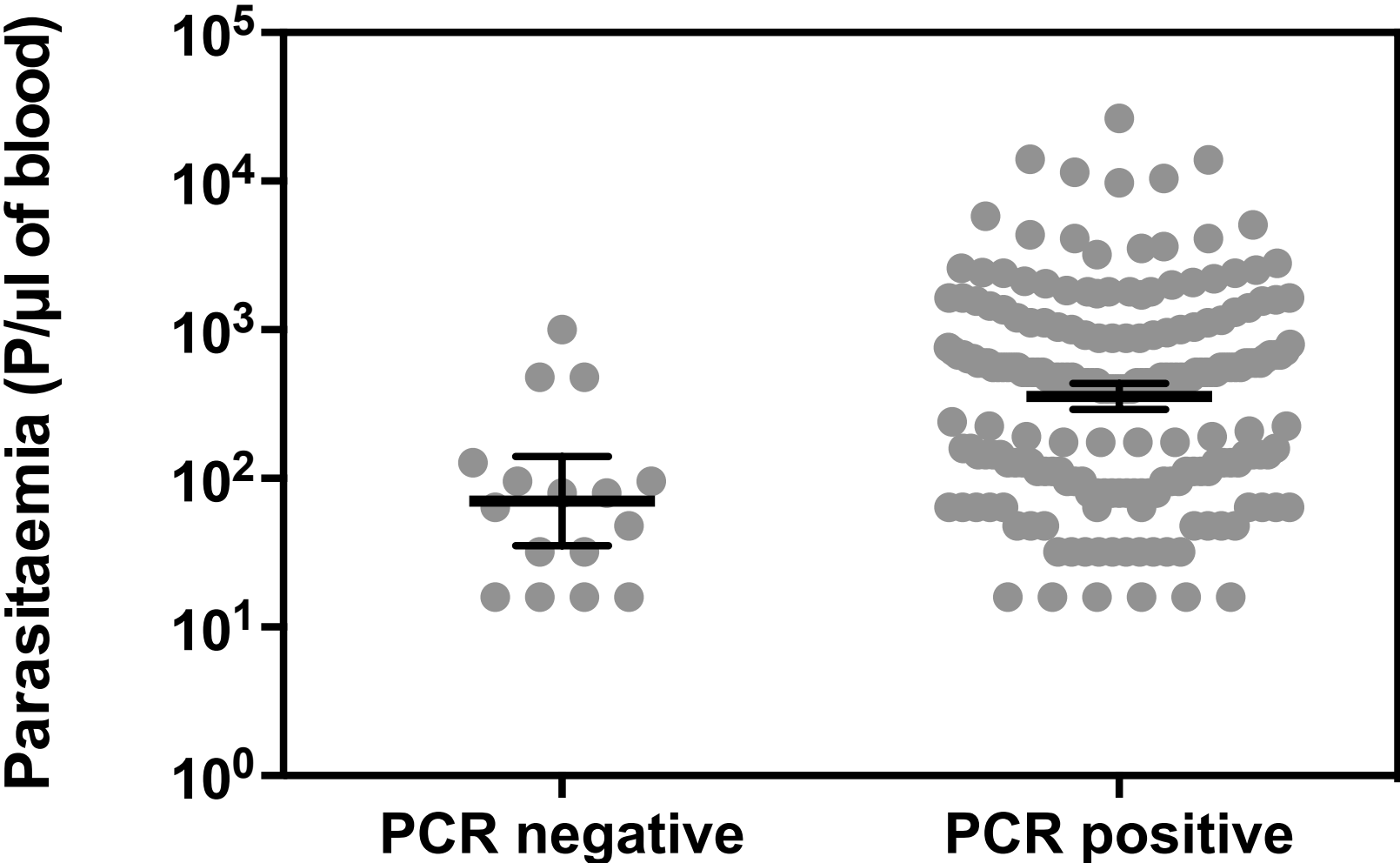

Supplement: Additional file 1 — PCR and microscopy derived prevalence. (A)P. falciparum detection by PCR and microscopy-based observation of thick smears. The overall higher prevalence detected by PCR (82.5% versus 78.8%) is due to 27 samples found positive by PCR and negative by microscopy, while 16 samples positive by microscopy were found negative by PCR. (B) Parasitaemia, as determined by microscopy, of the PCR negative (n = 16) and PCR positive (n = 214) samples. The geometric mean of the PCR negative samples is significantly lower than that of the PCR positive samples (70.3 versus 356.4, p < 0.01, unpaired one-tailed Welch’s t-test), suggesting slide reading errors or P. falciparum DNA degradation. [file 1475-2875-12-419-S1.pdf]

A

|          |      |       |       |       |
|----------|------|-------|-------|-------|
| n        | 372  | 371   | 274   | 267   |
| Average  | 71.3 | 109.5 | 112.7 | 137.3 |
| SD       | 2.0  | 3.4   | 2.6   | 2.3   |
| Max      | 74   | 118   | 118   | 143   |
| Min      | 63   | 98    | 102   | 130   |
| Expected | 70   | 108   | 115   | 137   |

B

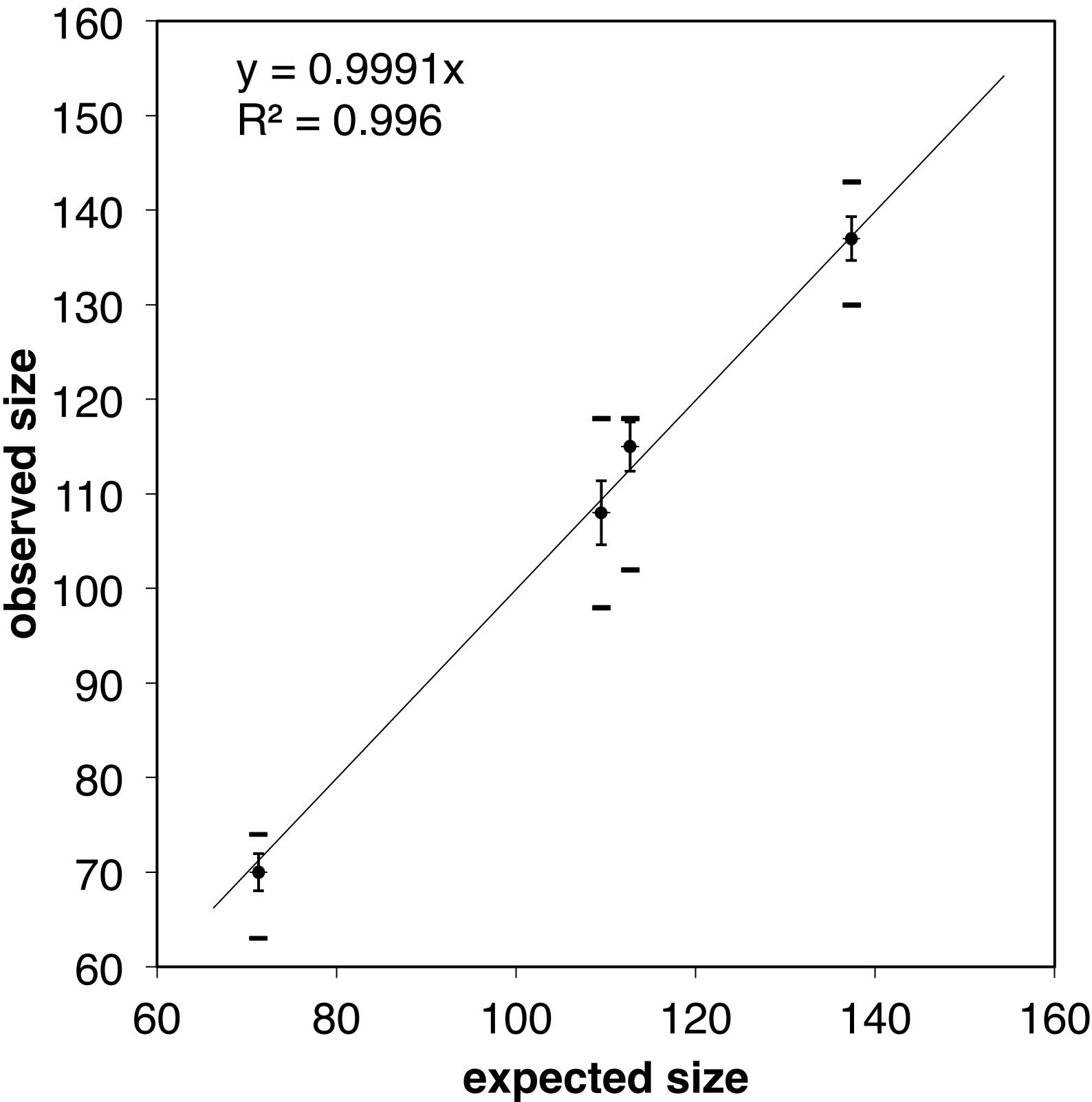

Supplement: Additional file 2 — msp2 genotyping precision. (A) Average, standard deviation and range of the observed sizes of fixed-size msp2 restriction digest products. (B) Linear regression between the observed and expected sizes of the fixed-size msp2 restriction digest products. The average, standard deviation, miminum and maximum value are reported. [file 1475-2875-12-419-S2.pdf]
