# Supplementary material for: Genetic diversity of Plasmodium falciparum among school-aged children from the Man region, western Côte d’Ivoire
Source: Malar J. 2013 Nov 15;12:419. doi: 10.1186/1475-2875-12-419 (PMC3842749; doi:10.1186/1475-2875-12-419)
Supplement: Additional file 3 — Relation between MOI and age. (A) Individual MOI grouped by age clusters (7 to 10 and 11 to 15 years old) in the four study villages. The red lines indicate the means and standard deviations. (B) The average MOIs are not significantly different in the two age clusters in each of the four study villages. [file 1475-2875-12-419-S3.pdf]

A

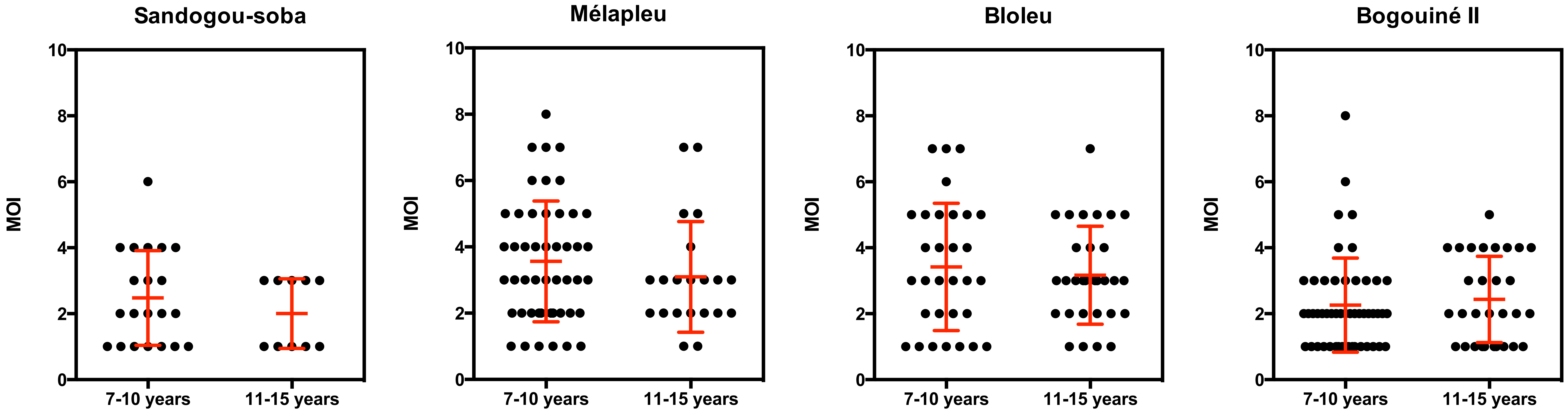

B

	Sandougou-soba	Mélapleu	Bloleu	Bogouiné II
MOI in 7-10 years (SD)	2.48 (1.44)	3.56 (1.82)	3.42 (1.93)	2.26 (1.43)
MOI in 11-15 years (SD)	2.00 (1.05)	3.10 (1.67)	3.17 (1.49)	2.43 (1.31)
p value (Mann-Whitney)	0.39	0.28	0.70	0.45
